# Effect of Different Prophylaxis Methods on Microleakage of Microfilled Composite Restorations

**DOI:** 10.5681/joddd.2012.014

**Published:** 2012-06-06

**Authors:** Soodabeh Kimyai, Narmin Mohammadi, Parnian Alizadeh Oskoee, Fatemeh Pournaghi-Azar, Mohammad Esmaeel Ebrahimi Chaharom, Melina Amini

**Affiliations:** ^1^Dental and Periodontal Research Center, School of Dentistry, Tabriz University of Medical Sciences, Tabriz, Iran; ^2^Associate Professor, Department of Operative Dentistry, School of Dentistry, Tabriz University of Medical Sciences, Tabriz, Iran; ^3^Assistant Professor, Department of Operative Dentistry, School of Dentistry, Tabriz University of Medical Sciences, Tabriz, Iran; ^4^Post-graduate Student, Department of Operative Dentistry, School of Dentistry, Tabriz University of Medical Sciences, Tabriz, Iran

**Keywords:** Microfilled composite resin, microleakage, prophylaxis methods

## Abstract

**Background and aims:**

This study was aimed at evaluating the effect of different prophylaxis methods on microleak-age of microfilled composite restorations.

**Materials and methods:**

In this in vitro study, class V cavities were prepared on buccal surfaces of 84 bovine teeth. The teeth were restored with Tetric N-Bond adhesive and Heliomolar composite resin. Subsequent to a thermocycling procedure and three months of storage in distilled water, the teeth were randomly assigned to four groups (n=21): (1) prophylaxis with a rubber cup and pumice; (2) prophylaxis with a brush and pumice; (3) prophylaxis with air/powder polishing device; and (4) no prophylaxis (the control group). Then the teeth were immersed in 2% basic fuchsin for 24 hours and sectioned for microleakage evaluation under a stereomicroscope. Data were analyzed using Kruskal-Wallis and Wilcoxon Signed Rankstests. Statistical significance was defined at p<0.05.

**Results:**

There were no statistically significant differences in occlusal and gingival microleakage between the groups (p=0.996 and p=0.860, respectively). In all the groups gingival margins exhibited significantly higher microleakage values compared to occlusal margins (p<0.0005).

**Conclusion:**

Prophylaxis methods had no adverse effect on marginal leakage of microfilled composite resin restorations.

## Introduction

Prophylaxis methods remove plaque and stain from tooth surfaces and depending on time and method might increase surface roughness and destroy superficial tooth structures.^1^ The surface finish of restorative materials can also deteriorate or even be destroyed by prophylaxis procedures, especially in class V restorations, because there is usually more plaque accumulation in cervical areas.^2^ Surface roughness of restorative materials can result in staining, accumulation of plaque, irritation of gingiva, and recurrent caries; it might also have a detrimental effect on the color and gloss of tooth-colored restorative materials. ^1, 2^

One of the most common methods used for tooth prophylactic procedures is application of pumice with a rotating rubber cup or a brush, which is often unsuccessful in removing stains and is time-consuming. As a result, more effective and time-saving techniques have been introduced, one of which is air/powder polishing device (APD) that operates by directing slurry of high-pressure water, air and sodium bicarbonate against the tooth surface.^3^ APD has been reported to be more efficient and time-saving, compared to rubber cup, in the removal of tooth stains and dental plaque.^3^ Some studies have revealed that APD removes large amounts of sound dentin and cement; in addition, surface changes have been observed in restorative materials.^3 , 4^

One important aspect of periodontal-restorative interaction is surface roughness of restorative materials.^2^ In this context, several studies have evaluated the effect of various prophylactic techniques on surface roughness of restorative materials, such as composite resins, glass-ionomers and giomers, with reports that the effect of prophylaxis methods varies depending on the restorative material used. It has been concluded that prophylaxis with pumice and brush resulted in a significantly higher surface roughness compared to prophylaxis with pumice and rubber cup in conventional glass-ionomers, compomers and giomers. Moreover, prophylaxis with APD resulted in a significantly higher surface roughening in comparison with two above-mentioned prophylaxis techniques in restorative materials.^5 - 7^

A similarly important aspect of periodontal-restorative interaction is marginal leakage of restorations.^2^ Gorfil et al did not report any detrimental effects on marginal leakage of amalgam and composite resin restorations with the use of 60-second ultrasonic cleaning and 120-second air-polishing techniques.^8^

Regarding the importance of relationship between dental restorations and periodontal health,^2^ and since the effect of different prophylaxis methods on microleakage of composite resin restorations has not been evaluated in previous studies, the present study was designed to evaluate the effect of three prophylaxis methods on microleakage of microfilled composite resin restorations.

## Materials and Methods

Eighty-four sound permanent bovine mandibular incisors^9^were used in this in vitro study. Visual examination and evaluation under a stereomicroscope (Nikon, Tokyo, Japan) did not reveal any cracks and structural defects in the teeth selected. The teeth were immersed in 0.5% chloramines T trihydrate solution for a week and then stored in distilled water in a refrigerator at 4ºC. Twenty-four hours before the study procedures, the teeth were conditioned in distilled water at 23±2ºC. Subsequently, class V cavities were prepared with the use of cylindrical diamond burs (Diatech Dental AG, Swiss Dental Instruments, CH-9435 Heerbrugg, Switzerland), measuring 3×3 mm in occlusogingival and mesiodistal dimensions with a depth of 2 mm on the buccal surface.^9^ The occlusal margin of each preparation was placed 1.5 mm coronal to the cemento-enamel junction (CEJ), with the gingival margin 1.5 mm apical to the CEJ.^1^Each bur was used for five cavity preparation procedures.^1^ No bevels were used on the margins; therefore, all the margins were butt-jointed.^12^ During preparation, all the tooth surfaces were kept wet to avoid dehydration. The cavity walls were etched ^11^with 37% phosphoric acid gel (N-Etch, Ivoclar Vivadent, Schaan, Liechtenstein) for 15 seconds,rinsed for 10 seconds and gently air-dried for 2 seconds in a manner to preserve dentin humidity. Then a one-bottle etch-and-rinse adhesive (Tetric N-Bond, Ivoclar Vivadent, Schaan, Liechtenstein) was applied according to manufacturer’s instructions and light-cured using Astralis 7 (Ivoclar Vivadent, Schaan, Liechtenstein) halogen light-curing unit for 20 seconds at a light intensity of 400 mW/cm^2^. All the cavities were restored with a microfilled composite resin (Heliomolar A2 shade, Ivoclar Vivadent, Schaan, Liechtenstein) using the incremental technique ^10^ in two 1-mm-thick layers. Each composite resin layer was cured for 20 seconds. Post-curing was carried out for 60 seconds at a light intensity of 700 mW/cm^2^. Subsequently, the specimens were finished and polished with diamond burs (Diamant Gmbh, D & Z, Goerzallee, Berlin, Germany) and polishing disks (Sof-Lex^ T ^, 3M ESPE, Dental Products, St. Paul, MN, USA), respectively. Then, the specimens were stored in distilled water at 37ºC for 24 hours,^13^ and thermocycled at 5±2ºC / 55±2ºC (500 times)^14^ with dwell and transfer times of 30 and 10 seconds using thermo-cycling machine (Vafaei Industrial Factory, Tehran, Iran), respectively.

The specimens were stored in distilled water at 37ºC for three months in an attempt to simulate the recall periods for maintenance therapy in the clinic.^6^ Then the teeth were randomly assigned to four groups (n=21):

Group 1: Buccal surfaces of the specimens were subjected to pumice-water slurry (Kemdent, Swindon, Wiltshire, UK) along with the use of a rotating rubber cup (Stoddard, Letchworth, Hertfordshire, UK) for 120 seconds in a slow-speed contra-angle handpiece at 2000 rpm. Each procedure was carried out with a new rubber cup.^5^

Group 2: The same procedure, as described for group 1, was carried out except that a rotating brush (Vericom Dental, Anyang, Gyeonggi, South Korea) was used instead of a rotating rubber cup.

Group 3: The specimen surfaces were treated with an air/powder polishing device (Air-Flow, Electronic Medical Systems, Nyon, Switzerland) for 120 seconds.^8^ The jet tip was positioned perpendicular to the surface at a distance of 10 mm. Regular paste was used during the first 60 seconds, which was replaced with a fine-particle paste for the next 60 seconds.

In groups 1-3, the specimens were rinsed under running water and further cleaned in an ultrasonic bath for 10 minutes after the prophylaxis methods.^5^

Group 4: Specimens in this group (control) did not undergo any prophylaxis methods.

Apices of teeth were sealed with utility wax in preparation for microleakage analysis. All tooth surfaces were covered with two layers of nail varnish, except for a 1-mm zone around the tooth-restoration interface. Subsequently, all the specimens were placed in 2% basic fuchsin for 24 hours.^1^ Finally, the teeth were divided in half buccolingually using a diamond disk (Diamont Gmbh, D&Z, Berlin, Germany) and evaluated under a stereomicroscope (Nikon, Tokyo, Japan) at ×16. Dye penetration was evaluated at occlusal and gingival margins using the following classification:^1^ 0: No dye penetration; I: Dye penetration along occlusal/gingival wall without encroaching the axial wall; II: Dye penetration along occlusal/gingival wall with axial wall involvement; III: Dye penetration on the entire axial wall and advancing toward the pulp.

### Statistical Analysis

Kruskal-Wallis test was used to compare occlusal and gingival microleakage between the groups. Wilcoxon Signed Ranks test was used to compare microleakage between occlusal and gingival margins in each group. Statistical significance was defined at P < 0.05.

## Results

Table 1 presents microleakage scores in the groups under study. There were no statistically significant differences in occlusal microleakage between the groups (P = 0.996). In addition, no significant differences were observed in gingival microleakage between the study groups (P = 0.860).

**Table 1:** Microleakage scores in the study groups

		Microleakage scores				
Groups	Margin	0	I	II	III	N
1 (pumice with rubber cup)	Occlusal	7	13	1	0	21
	Gingival	0	16	4	1	21
2 (pumice with brush)	Occlusal	7	12	2	0	21
	Gingival	0	15	4	2	21
3 (APD)	Occlusal	7	13	1	0	21
	Gingival	1	15	5	0	21
4 (without prophylaxis)	Occlusal	7	13	1	0	21
	Gingival	1	15	5	0	21

According to the results of the present study, there were statistically significant differences in occlusal and gingival microleakage scores between all the groups; gingival margins exhibited significantly more microleakage compared to occlusal margins (P <0.05).

## Discussion

In this study the effect of three prophylaxis methods on the microleakage of occlusal and gingival margins of microfilled composite resin restorations were evaluated. Microfilled composite resins are commonly used to restore cervical cavities due to their proper flexibility and low modulus of elasticity.^15^ Microleakage is usually evaluated by a dye penetration test and subsequent cutting of the specimens.^16 - 18^ Therefore, this technique was used in the present study.

The results of this study did not reveal any statistically significant differences in microleakage between the four groups and none of the prophylaxis methods had a detrimental effect on the marginal microleakage of composite resin restorations in comparison with the control group (without any prophylaxis method). In the same context, Gorfil et al reported that prophylaxis with an ultrasonic scaler and APD had no adverse effects on marginal leakage of class V amalgam and composite resin restorations.^8^ In contrast, Rajstein et al concluded that ultrasonic scaling had a detrimental effect on class V amalgam restoration surfaces and marginal integrity.^19^ The discrepancies between the results of the present study and a study by Rajstein might be attributed to differences in the prophylaxis methods used. In a previous study an ultrasonic scaler was used while in the present study APD and pumice were applied. It has been reported that APD results in more surface roughness in restorative materials compared to ultrasonic scaling. Sonic and ultrasonic scalers result in chips, scratches and loss of materials.^20^ In addition, ultrasonic techniques give rise to wider marginal gaps in class V amalgam restorations.^19^ Soares et al^21^ used a scanning electron microscope to evaluate the effect of periodontal treatment modalities on indirect composite restorations and reported that APD degrades cement line, creates porosities and an irregular surface and forms a gap at the tooth-restoration interface; in contrast, prophylaxis with pumice was reported to create grooves on the restoration and tooth surfaces.^21^ In a study carried out by Arabaci et al, no considerable cavities or craters were observed under a stereomicroscope on amalgam and composite resin samples after exposure to APD.^20^ Based on the results of a study by Rühling, the substance loss values (both in tooth structure and restorative materials) after polishing with polishing pastes were not significantly different from those observed with ultrasonic scaling plus polishing pastes; however, they were significantly greater with curettes plus polishing pastes due to higher sharpness of curettes.^2^ Differences in the results of studies might be attributed to instrumentation parameters, including application pressure, angle and time, device settings, materials tested (such as surface characteristics of materials) and differences in measuring techniques.^2^

Another finding of the present study was higher microleakage values in gingival margins compared to occlusal margins, consistent with the results of a previous study.^2^ Higher gingival margin microleakage values might be attributed to the organic content of dentin substrate and movement of tubular fluids in dentin, which might exert an influence on the bonding of etch-and-rinse adhesives to dentin. In addition, it has been reported that in class V cavities with gingival margins located 1-1.5 mm apical to CEJ dentinal tubules are oriented parallel to the cervical wall. Therefore, classic hybrid layer is not formed, which is another factor implicated in higher leakage values through gingival margins.^2^

The results of the present study showed that prophylaxis methods including pumice with rubber cup, pumice with brush and APD did not have any deleterious effect on marginal microleakage of microfilled composite resin restorations. However, according to the previous studies mentioned above, prophylaxis methods can give rise to surface roughening of restorative materials.^5 - 7 , 24^ Therefore, re-polishing of restorations subsequent to prophylaxis methods tested might be necessary.

It is suggested that the composite resin-tooth interface be evaluated by scanning electron microscopy and quality of margins of composite resin restorations be further evaluated using gap analysis in future studies.

## Conclusion

Within the limitations of this in vitro study it can be concluded that three prophylaxis methods investigated had no detrimental effects on marginal leakage of microfilled composite resin restorations.
